# Using ESTs to improve the accuracy of *de novo *gene prediction

**DOI:** 10.1186/1471-2105-7-327

**Published:** 2006-07-03

**Authors:** Chaochun Wei, Michael R Brent

**Affiliations:** 1 Laboratory for Computational Genomics and Department of Computer Science and Engineering, Washington University, One Brookings Drive, St. Louis, MO 63130, USA

## Abstract

**Background:**

ESTs are a tremendous resource for determining the exon-intron structures of genes, but even extensive EST sequencing tends to leave many exons and genes untouched. Gene prediction systems based exclusively on EST alignments miss these exons and genes, leading to poor sensitivity. *De novo *gene prediction systems, which ignore ESTs in favor of genomic sequence, can predict such "untouched" exons, but they are less accurate when predicting exons to which ESTs align. TWINSCAN is the most accurate *de novo *gene finder available for nematodes and N-SCAN is the most accurate for mammals, as measured by exact CDS gene prediction and exact exon prediction.

**Results:**

TWINSCAN_EST is a new system that successfully combines EST alignments with TWINSCAN. On the whole *C. elegans *genome TWINSCAN_EST shows 14% improvement in sensitivity and 13% in specificity in predicting exact gene structures compared to TWINSCAN without EST alignments. Not only are the structures revealed by EST alignments predicted correctly, but these also constrain the predictions without alignments, improving their accuracy. For the human genome, we used the same approach with N-SCAN, creating N-SCAN_EST. On the whole genome, N-SCAN_EST produced a 6% improvement in sensitivity and 1% in specificity of exact gene structure predictions compared to N-SCAN.

**Conclusion:**

TWINSCAN_EST and N-SCAN_EST are more accurate than TWINSCAN and N-SCAN, while retaining their ability to discover novel genes to which no ESTs align. Thus, we recommend using the EST versions of these programs to annotate any genome for which EST information is available.

TWINSCAN_EST and N-SCAN_EST are part of the TWINSCAN open source software package .

## Background

There are two major computational approaches to determining the exon-intron structures of genes: expression-based and *de novo*. Expression-based systems predict that a genomic nucleotide is exonic only if a transcript from it, or from a homologous gene (or a corresponding protein), has been sequenced. This approach can accurately predict genes whose transcripts have been sequenced and those that are highly similar to sequenced transcripts. However, its accuracy on genes that are not highly similar to sequenced transcripts is much lower [1,2]. This is a significant limitation, since sequencing cDNA libraries typically produces complete cDNA sequences from only about 50–60% of the genes in a genome. When genes that are partially covered by ESTs are included, that number may rise to 70–85%, depending on the depth of library sequencing and the complexity of the organism. Genes that are expressed at a low level or in a small number of tissues tend not to be sequenced even after sequencing libraries very deeply [3,4].

*De novo *gene prediction systems employ statistical models to predict gene structures using the sequences of one or more genomes as their only inputs. No cDNA sequences or other expression data are needed, so *de novo *methods can predict completely novel genes. However, they ignore the cDNA sequences that are available. As a result, they tend to be less accurate than expression-based methods on genes for which full-length cDNAs are available.

There is a long history of efforts to use databases of expressed sequences (ESTs, mRNAs, their conceptual translations, and experimental protein sequences) to enhance the accuracy of prediction systems that are based primarily on *de novo *methods. Studies that present quantitative evaluations of the effects of using ESTs alone, without using amino acid sequences from homologous genes, have reported mixed results [5-7]. Using a HMM-based *de novo *predictor, HMMGene, Krogh [7] reported no improvement in predictions for *Drosophila melanogaster*. Using GENIE, another HMM-based *de novo *predictor, Reese and colleagues reported a modest increase in sensitivity accompanied by a smaller decrease in specificity, also on *Drosophila *[6]. The best results were reported by Howe et al. [5]. Using GAZE, a generic evidence-combination framework, they obtained an increase in both the sensitivity and specificity of predictions by GeneFinder (P. Greene, unpublished) on *Caenorhabditis elegans*. Synthesizing these studies, it seems that better results were achieved by using a more stringent cutoff for similarity between the EST and the genome (93% identity for HMMGene, 95% for GENIE and GAZE). Better results were also achieved by using alignments created by EST_GENOME [8], a program designed to align ESTs with proper introns bounded by GT-AG (GAZE), rather than alignments created by BLASTN and then "fixed up" to make proper exons and introns (HMMGene and GENIE). Finally, better results were achieved on *C. elegans*, which has short introns and relatively less alternative splicing, than on *D. melanogaster*.

Another approach is to derive gene structures from a weighted combination of ESTs with multiple gene predictions, often including predictions from systems like ENSEMBL that use cDNA and protein alignments. This approach is exemplified by EuGene [9], Combiner [10], and its descendent JIGSAW [11]. However, with the exception of JIGSAW, none of the work described so far includes evaluations on mammalian genomes, which have long introns, many pseudogenes, and extensive alternative splicing. The JIGSAW publication includes evaluation on selected genes and regions from the human genome, but not on entire chromosomes.

The more successful of the methods outlined above work in part by boosting the scores of predicted introns that match intron gaps in EST alignments. For GENIE, the boost is large, "effectively constraining the system to ensure that the introns were correctly annotated according to the EST/cDNA evidence" [6]. For GAZE, the boost is a function of the EST alignment score: (%identity – 95) × length [5]. In neither case, however, is the EST scoring system trained automatically (Howe et al. reported that the automatic training method they tried did not work very well). Recently, several papers have reported success in training parameters for use of EST alignments, including EuGene [9], Combiner [10], and JIGSAW [11].

In this paper, we report on a new approach to integrating information from EST alignments with an HMM-based, *de novo *gene predictor. Rather than using fixed score boosts for compatible predictions, our method learns the degree to which a particular set of EST alignments is predictive of correct gene structure. This predictive power depends on the quality and quantity of the ESTs, the degree of alternative splicing, the alignment method, and the pre-processing method for filtering out questionable alignments. When used in combination with our state-of-the-art gene prediction programs, TWINSCAN and N-SCAN, this system can be automatically retrained to work well on both *C. elegans *and human. Furthermore, accuracy on genes or parts of genes without aligned ESTs is not compromised. On the contrary, genes without ESTs are predicted more accurately as a result of the constraints imposed by ESTs aligned to neighboring genes.

## Results

### Model for exploiting EST alignments

**Figure 1:**
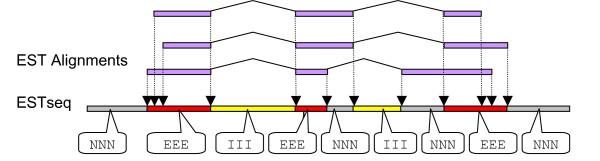
Construction of ESTseq from EST alignments. Each row of purple bars represents the aligned blocks of one EST, while the thin lines connecting the bars represent implied introns. The ESTseq representation contains an "E" for each base that is indicated as exonic (red), an "I" for each base that is indicated as intronic (yellow), and an "N" for each base that lies outside of all the alignments (gray). Regions that are indicated as intronic by some alignments and exonic by others are also labeled "N".

Our method for exploiting EST alignment information is very similar to the "conservation sequence" approach TWINSCAN uses to exploit genomic alignments [12,13]. First, all available EST sequences are aligned to the genome and alignments that fail certain quality criteria are filtered out (see Methods). Each nucleotide of the genome sequence is then assigned one of three symbols: **I **if it falls in an intron of all overlapping EST alignments, **E **if it falls in the exon (aligned region) of all overlapping EST alignments, and **N **if there is a disagreement among overlapping EST alignments or there are no overlapping EST alignments (Figure 1). The result is a sequence with one letter for each base of the input genome which represents much of the useful information in the EST alignments. We call this representation *ESTseq *by analogy to the conservation sequence or *conseq *that TWINSCAN uses for genomic alignments. Representing regions of disagreement among alignments in the same way as regions where no ESTs align allows the gene finder to rely on intrinsic information in the genome sequence when ESTs are inconclusive.

The EST sequence can be exploited by any HMM-based gene predictor. Each state of the HMM is required to emit both a target genome sequence and the corresponding ESTseq. When TWINSCAN uses ESTseq it emits ESTseq symbols, target genome bases, and conservation sequence symbols. Similarly, N-SCAN [14,15] emits ESTseq symbols together with columns of multi-genome alignments. All states must have probability models for the emission of ESTseq symbols, so these symbols can influence the likelihoods of functional annotations such as splice donor and acceptor, exon, intron, translation initiation and termination site, and so on. For example, the likelihood of emitting the **I **symbol from intron states should be greater than the likelihood of emitting **I **from exon states. Parameters for these models are estimated from examples of known gene structures together with their ESTseqs. See Methods for the ESTseq models we used in each HMM state.

### Accuracy evaluation: *C. elegans*

**Figure 2:**
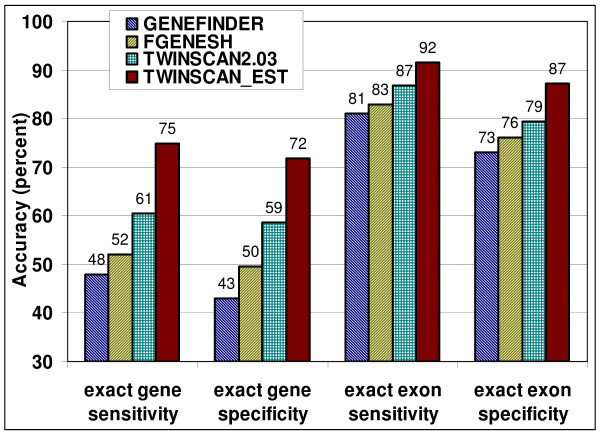
Results on the whole *C. elegans *genome (version WS130) using *C. briggsae *(version cb25.apg8) as the informant database and *C. elegans *ESTs from dbEST. The sensitivities are based on the 4,705 fully confirmed genes from WS130 and the specificities are based on those predictions that overlap with fully confirmed genes.

TWINSCAN_EST has been tested on two worm data sets. The first is the whole *C. elegans *genome (version WS130). *C. briggsae *version cb25.agp8 is used as the informant database. The results show 14% improvement in sensitivity and 13% in specificity in predicting exact gene structures compared to TWINSCAN 2.03, which does not use EST alignments (see Figure 2). TWINSCAN 2.03 was, in turn, significantly more accurate than both FGENESH (v. 1, with *C. elegans *parameters v.1) [16,17] and GENEFINDER (release 980504, P. Green, unpublished), the two most widely used ab initio gene prediction programs for nematodes. This difference is due, in part, to the fact that TWINSCAN uses comparison to the *C. briggsae *genome, while the others do not [18]. (For a discussion of sensitivity and specificity estimates using incomplete annotation sets, please see [13]).

**Figure 3:**
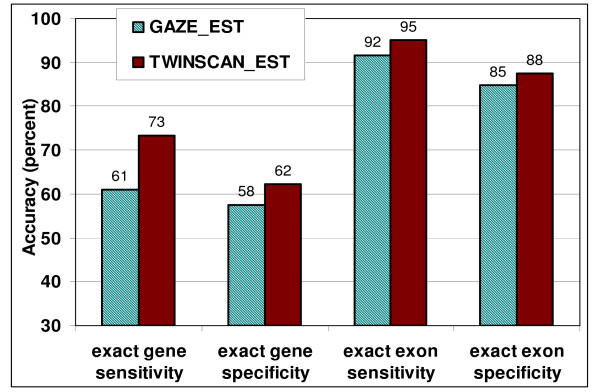
Accuracy on GAZE merged data set. Both GAZE_EST and TWINSCAN_EST used the same BLAT alignments of *C. elegans *ESTs from dbEST (1/20/2005). Informant database for TWINSCAN_EST is the *C. briggsae *genome (version cb25.apg8). 305 of the 325 gene loci have at least one EST alignment.

The second test used the 2 Mb GAZE dataset, which was created by concatenating the sequences of 325 genes flanked by half the intergenic region to the closest known gene on each side [5]. *C. elegans *ESTs were downloaded from dbEST (1/20/2005) [19], aligned to the GAZE genomic sequence by using BLAT, and filtered for alignment quality (Methods). Both GAZE_est and TWINSCAN_EST were run on the same genomic sequence with the same EST alignments. The results show that TWINSCAN_EST is more accurate than GAZE_est, especially for exact gene structure prediction (Figure 3). TWINSCAN_EST has 73% gene sensitivity and 62% gene specificity compared to GAZE_est's 61% and 58%.

Although TWINSCAN_EST shows substantial improvement over previous systems when evaluated against fully confirmed worm genes, these genes are more likely to have aligned ESTs than a randomly selected gene. Thus, an independent test is needed to determine how TWINSCAN_EST would perform on genes with no aligned ESTs. We carried out such a test by running it on the entire genome with an empty EST database, so that no gene had aligned ESTs. This resulted in slight improvements to sensitivity and specificity in exact gene prediction compared to predictions by TWINSCAN 2.03, which does not consider the presence or absence of ESTs (Table 1). These improvements may result from applying a slight score penalty to exons and genes without ESTs – in this case all exons and genes. Since the training set includes genes with EST evidence, a region without EST alignment will be considered more probable outside a gene region than in a gene region. Such a penalty would eliminate predicted exons and genes with marginal scores, in effect filtering out the lowest scoring predictions from TWINSCAN 2.03. Since the lowest scoring predictions are mostly incorrect, this would improve accuracy. On the other hand, the improvement in gene accuracy is small, and exon sensitivity does not improve, so it is safe to conclude that novel genes with no ESTs are predicted with approximately the same accuracy by TWINSCAN_EST and TWINSCAN 2.03.

The previous experiment in which all ESTs were deleted from the database may yield an overly pessimistic assessment of TWINSCAN_EST's accuracy on novel genes with no aligned ESTs. It is possible that the presence of EST alignments for some genes may improve the accuracy of TWINSCAN_EST on the neighboring genes even when those neighboring genes have no aligned ESTs. The intuition is that certain kinds of mistakes, such as incorrectly splitting a gene with an EST and joining part of it to a neighbor without an EST, will become much less common. To test whether such indirect benefits actually exist, we did a partial EST deletion experiment. All fully confirmed WS130 genes were divided into 10 groups at random, each containing about 10% of the fully confirmed genes. One group of fully confirmed genes was selected, its ESTseq was masked with "N", and TWINSCAN_EST was run on the entire genome. These steps were repeated 10 times. Each time, the ESTseq for a different 10% of the confirmed genes was masked, so that the ESTseq for each confirmed gene was masked in exactly one repetition. We then computed the average accuracy statistics over the 10 runs for both the masked and unmasked genes. Results are shown in Table 1. The gene sensitivity of TWINSCAN_EST on the genes with masked ESTseq was 2.4% higher than TWINSCAN 2.03 and the specificity was 1.9% higher. In addition, exon and gene accuracy were higher than TWINSCAN_EST with blank EST sequence, indicating that the presence of ESTs for other genes did indeed improve the accuracy of genes with no ESTs.

The previous experiments show TWINSCAN_EST's accuracy on genes with or without aligned ESTs. In practice, many genes are partially covered by ESTs. To investigate the effect of partial EST coverage, we did the following experiment. ESTseqs were generated as in the TWINSCAN_EST experiment for Figure 2. The ESTseq for each fully confirmed WS130 gene was then N-masked over a contiguous, randomly chosen 50% of its genomic extent (see Methods). The predictions were evaluated on all the confirmed genes. The gene sensitivity was 69%, which is about halfway between the gene sensitivity of TWINSCAN 2.03 (61%) and TWINSCAN_EST without ESTseq masking (75%). The gene specificity is 67%, which is about two-thirds of the way from that of TWINSCAN 2.03 (59%) to that of TWINSCAN_EST without ESTseq masking (71%).

### Accuracy evaluation: human

**Figure 4:**
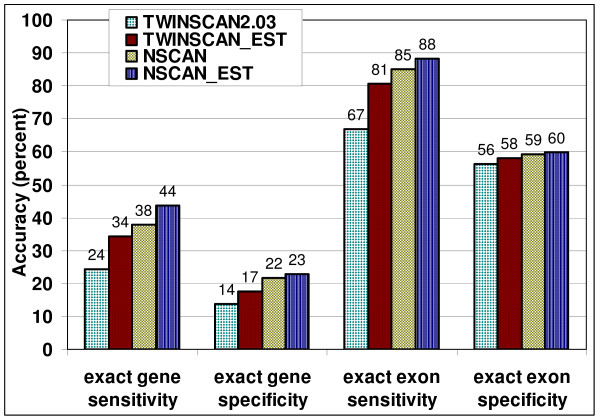
Accuracy of TWINSCAN, TWINSCAN_EST, NSCAN and N-SCAN_EST on the human genome. For TWINSCAN and TWINSCAN_EST, the mouse genome sequence is used as the informant database. For NSCAN and N-SCAN_EST, mouse, rat and chicken genomes are used as the informant databases. Human ESTs are from dbEST. For all methods, pseudo genes are masked out first [41].

TWINSCAN_EST and N-SCAN_EST were also tested on the human genome (NCBI Build 35). On this dataset, TWINSCAN_EST produced about 10% improvement in sensitivity and 3% in specificity in predicting exact gene structures compared to TWINSCAN 2.03 (see Figure 4). N-SCAN_EST produced a 6% improvement in sensitivity and 1% in specificity on exact gene structure level compared to N-SCAN. Approximately 36% of genes in our RefSeq-based annotation have a transcript with a spliced 5' UTR. For those that do, the sensitivity and specificity of N-SCAN (without ESTs) is similar to its sensitivity and specificity on genes without a spliced 5'UTR. However, N-SCAN_EST performs better on genes without a spliced 5'UTR than on those with a spliced 5'UTR by 3.5% in gene sensitivity and 5% in gene specificity. On genes with a spliced 5' UTR, N-SCAN_EST produced a 3.5% improvement in sensitivity and 1.4% in specificity as compared to N-SCAN without ESTs.

While this paper was in revision, a paper was published describing AUGUSTUS+, a new, trainable system capable of combining evidence from EST alignments with *de novo *gene prediction [20]. We compared the accuracy of N-SCAN_EST and AUGUSTUS+ by running them on human chromosome 22 using the same EST alignments (see Methods). Comparing the results to aligned RefSeq genes, N-SCAN_EST's sensitivity and specificity for predicting the exact ORFs were 47% and 24%, respectively. The comparable numbers for AUGUSTUS+ using the same EST alignments were 38% and 19%, respectively.

### Impact of training EST parameters

**Figure 5:**
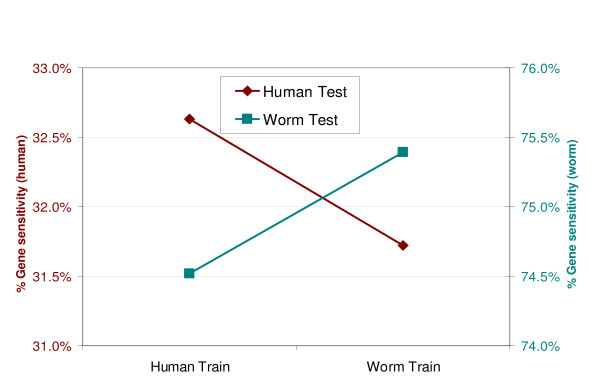
Trainability of ESTseq parameters. The human and worm genes were each divided into two halves, one for training and one for testing. ESTseq parameters were estimated separately from half the human genes and half the worm genes. Each set of parameters was then tested separately on the other half of the human genes and the other half of the worm genes. The same models were used for both human and worm ESTseqs (5^th^-order Markov Models for the coding regions, UTRs, Introns and intergenic regions, 43-base-long 2^nd^-order WAM for splice acceptor sites and 9-base-long 2^nd^-order WAM for the splice donor sites).

One of the differences between the ESTseq approach and most previous approaches is that our system can be trained, using known gene structures, to take advantage of the unique characteristics of a particular set of EST alignments to a particular genome. To test the effects of training on accuracy, we first performed cross-validation training for TWINSCAN_EST for human on human EST alignments and TWINSCAN_EST for *C. elegans *on *C. elegans *EST alignments (see Figure 5). Next, we swapped the ESTseq parameters of the systems trained for human and worm. The effect of training on accuracy was modest but clear – gene sensitivity is greater when a system trained for worm ESTs is used on worm ESTs and a system trained for human ESTs is used on human ESTs (Figure 5). Applying either one of the EST parameter sets to both species results in lower accuracy. The same pattern of results is seen for gene specificity (data not shown).

### Impact on an annotation pipeline using full length cDNA sequences

A complete pipeline for predicting exon-intron structures must give precedence to full length cDNA alignments over all other sources of evidence. The degree to which such a pipeline relies on ESTs and *de novo *gene prediction depends on how extensive is the set of available full length cDNAs. For example, we recently built a system in which the first stage is aligning full-ORF cDNA sequences to their native locus using our new cDNA-genome aligner, Pairagon [21]. The CDS GenBank annotations of the cDNA sequences were used to convert these alignments into gene structures. Where there is no full-length cDNA to align, we used N-SCAN_EST together with ESTseq created from BLAT alignments. This system was independently evaluated on the human ENCODE regions as part of the recent EGASP community evaluation [22,23] and found to be comparable in accuracy to the ENSEMBL pipeline (slightly better by most measures).

**Figure 6:**
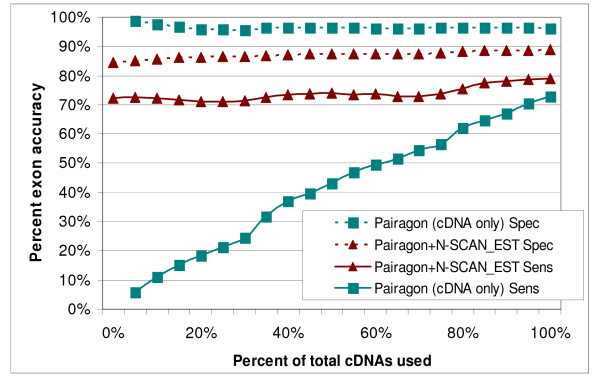
Accuracy of Pairagon cDNA alignments alone compared to Pairagon+N-SCAN_EST as a function of the number of cDNAs used. A total of 445 cDNAs aligned to the 31 human ENCODE test regions. The *x *axis shows the percentage of these 445 that were used. From left to right, 5% of unused cDNAs were randomly picked and added to those used previously.

In order to investigate the contribution of N-SCAN_EST to the Pairagon+N-SCAN_EST pipeline, we compared the sensitivity and specificity of Pairagon's cDNA alignments alone to that of the entire pipeline with N-SCAN_EST, at various levels of cDNA coverage. Accuracy at the exon level is plotted in Figure 6 (gene level results are qualitatively similar). The specificity of both systems is independent of cDNA coverage. As expected, including N-SCAN_EST predictions decreases specificity somewhat. However, including N-SCAN_EST predictions increases the sensitivity approximately as much as it decreases specificity, even at the maximum level of cDNA coverage, resulting in an even trade-off. As cDNA coverage decreases, the tradeoff favors the combined system more and more. The sensitivity of the cDNA-only system declines linearly with the number of input cDNAs, whereas the sensitivity the combined system remains high even when cDNA coverage is very low.

## Discussion

Our method for integrating information from EST alignments with an HMM-based gene predictor has four key features:

1) It can be trained to take advantage of the statistical characteristics of specific sets of EST alignments.

2) It substantially improves the accuracy (both sensitivity and specificity) of gene prediction on genes that have aligned ESTs.

3) It improves accuracy on genes that do not have aligned ESTs when they are interspersed with genes that do.

4) It predicts genes at least as accurately as the pure-HMM-based predictors when no ESTs align to the target genome.

Thus, the use of EST information comes at no cost. TWINSCAN_EST and N-SCAN_EST have the key benefit of a *de novo *gene finder – namely, the ability to find completely novel genes without sequence similarity to known genes – yet they are more accurate on genes for which EST information is available. Compared to other *de novo *gene finders, TWINSCAN is the most accurate program available for nematodes [18]. Likewise, N-SCAN is the most accurate *de novo *predictor available for mammals as measured by exact CDS gene prediction and exact exon prediction [15,18,24]. Other programs are either more specific but less sensitive (EXONIPHY) [25] or more sensitive but less specific (AUGUSTUS-dual, Stanke, unpublished) in predicting individual coding nucleotides. Thus, we would recommend using the EST versions of these programs on any genome for which there is EST information.

We also showed that combining N-SCAN_EST with a state-of-the-art system for aligning full length cDNAs yields a pipeline whose exon-prediction accuracy shows relatively little dependence on the number of available cDNA sequences. Thus, low cost EST sequencing can be substituted for expensive sequencing of full length cDNAs with limited accuracy reduction.

The real goal of gene prediction is not to find known genes but to find novel genes that can be verified experimentally. N-SCAN_EST has proven very useful in this regard. As part of an ongoing project we are using RT-PCR and sequencing to obtain novel human cDNA sequences. In these experiments, we target predicted introns with at least one splice site that is not in a region previously known to be transcribed – that is, not in an intron or exon defined by the alignment of any human mRNA or EST. By targeting predictions from N-SCAN_EST, we have verified more than a thousand novel introns. Thus, in addition to its application for annotating genomes with few full length cDNAs, N-SCAN_EST is also useful for well-studied genomes like that of *Homo sapiens*.

## Conclusion

TWINSCAN_EST and N-SCAN_EST are more accurate than TWINSCAN and N-SCAN, while retaining their ability to discover novel genes to which no ESTs align. Thus, we recommend using the EST versions of these programs to annotate any genome for which EST information is available.

## Methods

### ESTseq models for each state

In our implementation, the ESTseq models are homogeneous Markov chains for UTR, intron, and coding states, and position-specific Markov chains (sometimes called WAMs) for donor and acceptor site models.

### Procedure for masking 50% of the ESTseq for each gene

Let [0, 1] stand for a gene region. A random number *a *in the range [0, 1] is generated, then all ESTseq bases in the region [*a*, *a*+0.5] were masked with "N" if *a*< = 0.5 or bases in region [*a*, 1] U [0, *a*-0.5] were masked with "N" if *a*>0.5. As a result, at least 50% of each gene region was not covered with any EST alignment. TWINSCAN_EST was then run on the entire genome with these masked ESTseqs.

### Sequences

The *C. elegans *genome sequence version WS130 was downloaded from the WormBase website [26-28]. The *C. briggsae *genome sequence version cb25.agp8 was downloaded from the Sanger Institute [29]. Approximately 300,000 *C. elegans *ESTs were downloaded from dbEST (1/20/2005 version) [19,30]. The genome sequence for the GAZE dataset was downloaded from the GAZE website [31]. The informant database (*C. briggsae*) and EST database for the GAZE dataset were the same as for the whole *C. elegans *genome WS130.

Human ESTs were downloaded from dbEST on January 20^th ^2005. The informant database for TWINSCAN is the mouse genome [32] Build 33 (mm5 on the UCSC browser). Other informant datasets for N-SCAN include mouse, rat [33] (UCSC rn3) and chicken [34] (UCSC Galgal2) genomes [14,15].

### Genome alignments

For worm datasets, conservation sequences were generated from WU-BLAST [35] alignments of the whole *C. elegans *genome against the *C. briggsae *genome. First, *C. briggsae *sequences longer than 150 kb were cut into 150 kb sequences with 20 kb overlap, and then the Blast database was generated from all sequences after they had been masked by NSEG with default parameters. BLASTN parameters were "M = 1 N = -1 Q = 5 R = 1 B = 10000 V = 100 lcfilter filter = seg filter = dust topcomboN = 1".

The human chromosomes were split into 1 Mb fragments first, and then conservation sequence was constructed for each fragment.

### ESTseqs

*C. elegans *ESTs were aligned to WS130 by using stand alone version 25 of BLAT [36]. ESTseqs were generated using only those EST alignments in which the number of matches was at least 95% of the length of the entire EST, including unaligned portions. These alignments were projected onto genomic sequence to generate ESTseq as shown in Figure 1. For the GAZE dataset, similar procedures were done.

Human ESTs were aligned to the whole human genome by BLAT. An alignment was included only if its number of matches was at least 98% of the length of the entire EST. Those selected alignments were projected to the genomic sequence to generate ESTseqs as shown in Figure 1. The ESTseq of each chromosome was then split into 1 Mb fragments corresponding to the genomic sequences.

### ESTseq parameter estimation

ESTseq parameter estimation is similar to conservation sequence parameter estimation. Given ESTseqs and the corresponding gene structures, distinct sets of parameters are estimated for the coding regions (excluding translation initiation and termination signals), UTRs, intron states, donor and acceptor splice site signals, and translation initiation and termination signals. For TWINSCAN_EST on *C. elegans*, 1^st^-order Markov chains were used for coding, UTR, intron states, and the translation initiation and termination signals. A 43-base-long, 2^nd^-order WAM was used for acceptor splice site signals and 9-base-long, 2^nd^-order WAM was used for donor splice site signals. Regions between 1000 bases and 150 bases upstream of the start of translation and downstream of the stop of translation were used as intergenic regions. Intergenic regions' ESTseqs were used as the null model for each state.

For N-SCAN_EST on human, the single 5' UTR state in TWINSCAN is replaced by four 5' UTR states. Those states are: a) unspliced UTR from transcription start site (TSS) to the translation start site; b) initial noncoding exon (from the TSS to the splice donor); c) internal noncoding exon (from acceptor to donor) and d) the noncoding segment of the exon from acceptor splice site to the start of translation [see 14 for details]. 5^th^-order Markov models were used for all ESTseq models except the acceptor and donor splice site models, which were the same as for worm.

When 5^th ^order models are used for the worm data, as for human, all accuracies are within a fraction of a percent of those reported in this paper.

For training and evaluation purpose, human RefSeq mRNAs excluding the predicted XM_ accessions [37-39], aligned to human genome Build 35/hg17 were downloaded from the UCSC genome browser [40]. The RefSeq annotation was then cleaned by removing genes with in frame stop codons. There were 17,798 transcripts remaining, 17,120 of which contain UTR annotations. In order to estimate the ESTseq parameters, single-gene ESTseqs were cut out from the whole chromosome ESTseq with an additional 1000 bases on each end as intergenic regions. Parameters were estimated from these single-gene ESTseqs and the corresponding gene structures.

### N-SCAN_EST and Augustus+ comparison on human chromosome 22

In order to do a fair comparison to AUGUSTUS+, BLAT alignments of all spliced human ESTs on human chromosome 22 (Build 35/hg17) were downloaded from the spliced human EST track in the UCSC genome browser [40] on March 12^th^, 2006. These EST alignments were input into both Augustus+ and N-SCAN_EST. EST parameters for N-SCAN_EST were estimated from the cleaned RefSeq annotations on chromosome 1, 2, 20 and 21. EST Parameters for Augustus+ were estimated by its author from chromosome 21.

### Result evaluation

For the WS130 dataset, TWINSCAN_EST's performance was tested by 8-fold cross validation. The whole genome was split into fragments of about 500 kb. Each fragment was randomly assigned to one of the eight groups. TWINSCAN_EST was trained on fully confirmed genes from seven of the eight groups and run on the fragments from the eighth group to avoid training and testing on the same data set. For TWINSCAN_EST on the GAZE data set, no cross validation was applied. Parameters were estimated from all fully confirmed genes of WS130.

## Figures and Tables

**Table 1:** Results for deletion experiment. The first column is for TWINSCAN 2.03 and the remaining 3 columns are for TWINSCAN_EST. The second column is for the TWINSCAN_EST performance with empty ESTseq, i.e., all bases in ESTseqs are 'N's. For the third and fourth column, 10% of genes in the annotation were set to "N"s. The third column is for TWINSCAN_EST's performance on the 10% of genes with masked ESTseqs and the last column is for the 90% of genes with unmasked ESTseqs. Results show that EST alignments improve the prediction accuracy and do not compromise the capability to predict novel genes where EST alignments do not exist (column 2). Specificities are based on predictions that overlap with annotations by at least 1 bp.

	**TWINSCAN2.03**	**TWINSCAN_EST**		
		
		**Blank ESTseq**	**10% with ESTseq masked**	**90% with ESTseq unmasked**
Gene_sn	60.6	61.3	63.0	74.7
Gene_sp	58.6	59.8	60.5	71.5
Exon_sn	86.9	86.2	86.4	91.5
Exon_sp	79.5	80.8	81.1	87.0
